# Pharmacokinetics and Optimal Dosing of Phenobarbital

**DOI:** 10.15844/pedneurbriefs-32-7

**Published:** 2018-09-05

**Authors:** Safiullah Shareef, Murtaza N. Ali

**Affiliations:** 1 Texas Child Neurology, Plano, TX; 2 University of North Texas Health Science Center, Fort Worth, TX

**Keywords:** Epilepsy, Phenobarbital, Antiepileptic drugs

## Abstract

Researchers from the Baylor College of Medicine in Houston, TX, USA, conducted a *retrospective population pharmacokinetic* analysis of 355 patients ages less than 19 years of age in the inpatient setting who were initiated on intravenous or oral phenobarbital therapy and had one or more serum phenobarbital concentrations sampled.

Researchers from the Baylor College of Medicine in Houston, TX, USA, conducted a *retrospective population pharmacokinetic* analysis of 355 patients ages less than 19 years of age in the inpatient setting who were initiated on intravenous or oral phenobarbital therapy and had one or more serum phenobarbital concentrations sampled. The data collected from these patients was used to develop a virtual patient, one for every developmental stage (neonatal, infant, child, and adolescent). Each of the virtual patients had 10,000 simulations run on it for various dosing strategies (10, 20, 30, and 40 mg/kg) to determine an optimal intravenous phenobarbital loading dose that would achieve a serum concentration of 20-40 mg/L at 2 hours post-dose. Using the optimal loading dose, the simulation was performed for oral phenobarbital dosing to maintain a trough concentration of 20-40 mg/L after 7 days of therapy. The study noted that phenobarbital dosing should take into account the patient’s fat-free mass, postmenstrual age, serum creatinine, and age in years. Phenobarbital pharmacokinetics were not affected by body temperature. The study concluded that an intravenous dose of 30 *mg kg* ^−1^ once followed by 4-6 *mg kg* ^−1^ *d* ^−1^ divided twice daily resulted in therapeutic serum concentrations at 7 days. Also, the drug–drug interactions of midazolam, phenytoin, and pantoprazole significantly affected phenobarbital concentrations. [1]

COMMENTARY. Phenobarbital remains the first line drug therapy for the treatment of suspected neonatal seizures. Despite its extensive use in the neonatal population, the pharmacokinetics of phenobarbital have not been quantified across the pediatric age spectrum [2].

There are many factors that can influence dosing in this patient group including bioavailability and drug-drug interactions. The bioavailability of Phenobarbital has been documented at ~90% in adults and shown to be much less in neonatal populations [1]. It is hypothesized that growth-related changes in organ function and maturation of enzyme systems used to metabolize phenobarbital (2C19 subunit of the CYP Enzyme, matures significantly in first 6 months of life) play a factor. These changes and developments in neonates can make it difficult to correctly dose and maintain therapeutic dose of phenobarbital in neonates.

The study recommends using postmenstrual age in patients younger than 2 years of age to account for changes in clearance over the neonatal and infant age period. The study also reconfirmed that pantoprazole increased phenobarbital clearance and phenytoin decreased its clearance. Unique to this study was a new finding that midazolam decreased phenobarbital clearance [1]. The study made no comment on morphine or opioid receptor based medications and their effect on phenobarbital. Although, the authors did recognize that the percentage of patients within the study population on multiple interacting medications was small (<10%).

Based on the simulated data the authors notably suggest using a higher loading dose (30 *mg kg* ^−1^ IV) than their institutional guidelines (10-20 *mg kg* ^−1^ IV loading dose). Fat-free mass was used in their calculations instead of the patient’s actual weight. They also had different recommendations on the daily maintenance dosing: 4-6 *mg kg* ^−1^ *d* ^−1^ to be divided twice daily. Despite phenobarbital having a long half-life, the simulation suggests twice daily dosing may prevent low trough levels.

## Disclosures

The author(s) have declared that no competing interests exist.

## References

[1] Moffett BS, Weingarten MM, Galati M, Placencia JL, Rodman EA, Riviello JJ (2018). Phenobarbital population pharmacokinetics across the pediatric age spectrum. Epilepsia.

[2] Quinlan SM, Rodriguez-Alvarez N, Molloy EJ, Madden SF, Boylan GB, Henshall DC (2018). Complex spectrum of phenobarbital effects in a mouse model of neonatal hypoxia-induced seizures. Sci Rep.

